# 1,1,2,2-Tetra­kis(di-*o*-tolyl­phosphino)ethane

**DOI:** 10.1107/S1600536809032942

**Published:** 2009-08-26

**Authors:** Elizabeth M. Sisler, Karla Arias, Danielle L. Gray, Quinetta D. Shelby

**Affiliations:** a DePaul University, Department of Chemistry, 1110 West Belden Avenue, Chicago, Illinois 60614, USA; b University of Illinois, School of Chemical Sciences, Box 59-1, 505 South Mathews Avenue, Urbana, Illinois 61801, USA

## Abstract

The complete molecule of title compound, C_58_H_58_P_4_, is generated by a crystallographic twofold rotation axis that passes through the center of the C(methine)—C(methine) bond of length 1.582 (4) Å. The C—P bond lengths are 1.8824 (19) and 1.8991 (19) Å. The P—C—P angle of 109.69 (9)° is essentially equal to the expected value of 109.5° for a tetra­hedral C atom. Although the C(methine)—P—C(aromatic) bond angles range from 102.67 (9) to 107.04 (9)°, the C(aromatic)—P—C(aromatic) bond angles of 96.72 (9) and 97.29 (9)° are significantly smaller. The steric demands of the *o*-tolyl groups cause deviations from the bond lengths and angles reported for its phenyl analog.

## Related literature

For 1,1,2,2-tetra­kis[(diphen­yl)phosphino]ethane, see: Braunstein *et al.* (1995*a* ▶). For oxidative coupling of (bis­phosphino)methanides, see: Braunstein *et al.* (1995*b* ▶); Schmidbaur & Deschler (1983 ▶). 
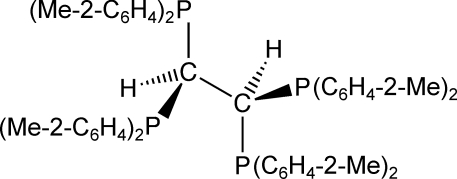


## Experimental

### 

#### Crystal data

C_58_H_58_P_4_
*M* *_r_* = 878.92
Monoclinic, 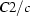

*a* = 21.8875 (11) Å
*b* = 10.9702 (6) Å
*c* = 19.691 (1) Å
β = 90.761 (3)°
*V* = 4727.6 (4) Å^3^
*Z* = 4
Mo *K*α radiation
μ = 0.20 mm^−1^
*T* = 193 K
0.42 × 0.40 × 0.24 mm

#### Data collection

Bruker Kappa APEXII CCD diffractometer
Absorption correction: multi-scan (*SADABS*; Bruker, 2005 ▶) *T*_min_ = 0.942, *T*_max_ = 0.976
19717 measured reflections
4345 independent reflections
3343 reflections with *I* > 2σ(*I*)
*R*_int_ = 0.049

#### Refinement

*R*[*F* ^2^ > 2σ(*F* ^2^)] = 0.039
*wR*(*F* ^2^) = 0.102
*S* = 1.01
4345 reflections
284 parameters
H-atom parameters not refined
Δρ_max_ = 0.35 e Å^−3^
Δρ_min_ = −0.19 e Å^−3^

### 

Data collection: *APEX2* (Bruker, 2004 ▶); cell refinement: *SAINT* (Bruker, 2005 ▶); data reduction: *SAINT*, *XPREP* (Bruker, 2005 ▶) and *SADABS* (Bruker, 2007 ▶); program(s) used to solve structure: *SHELXTL* (Sheldrick, 2008 ▶); program(s) used to refine structure: *SHELXTL*; molecular graphics: *SHELXTL* and *CrystalMaker* (*CrystalMaker*, 1994 ▶); software used to prepare material for publication: *XCIF* (Bruker, 2005 ▶).

## Supplementary Material

Crystal structure: contains datablocks I, global. DOI: 10.1107/S1600536809032942/ng2629sup1.cif

Structure factors: contains datablocks I. DOI: 10.1107/S1600536809032942/ng2629Isup2.hkl

Additional supplementary materials:  crystallographic information; 3D view; checkCIF report

## Figures and Tables

**Fig. 1.**
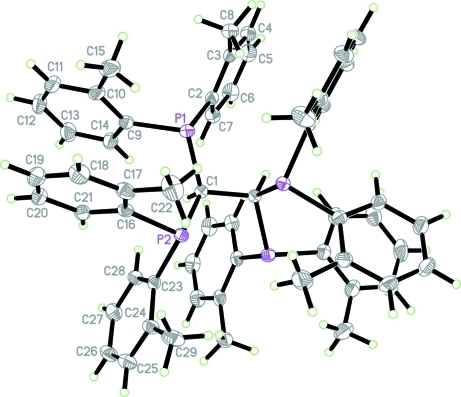
Molecular structure of the title compound showing 35% probability ellipsoids for non-H atoms and circles of arbitrary size for H atoms. The unlabeled atoms are related to the labeled atoms by the symmetry operator (1 - x, y, 0.5 - z).

## supplementary crystallographic information

### Comment

The title compound, [*P*(*o*-tolyl)_2_]_2_CH—CH[*P*(*o*-tolyl)_2_]_2_, was unintentionally obtained as one of two isomeric products from the reaction of Li{CH[*P*(*o*-tolyl)_2_]_2_} with PdCl_2_. It is formed by the C—C cross-coupling of two CH[*P*(*o*-tolyl)_2_]_2_ units, whereas its isomer is a P—C coupling product. Oxidative coupling reactions involving (bisphosphino)methanide compounds have been reported (Braunstein *et al.*, 1995*a*; Braunstein *et al.*, 1995*b*; Schmidbaur & Deschler, 1983).

The title compound crystallizes in *C*2/*c*, whereas the space group is *P*2_1_/*n* for its phenyl analog, [P(Ph)_2_]_2_CH—CH[P(Ph)_2_]_2_ (Braunstein *et al.*, 1995*a*). The structure of the title compound has a twofold rotation axis through the center of the C(methine)–C(methine) bond (Fig. 1). The atomic positions of only one CH[*P*(*o*-tolyl)_2_]_2_ unit were determined, and the atomic positions of the other unit are related by the symmetry operator (1 - *x*, *y*, 0.5 - *z*). The methine hydrogen atoms adopt a *gauche* conformation that likely maximizes the *π* interactions of the aromatic rings. The C1(methine)—C1(methine) bond length is 1.582 (4) Å, and its C1—P bond lengths are 1.8824 (19) and 1.8991 (19) Å. The P1—C1—P2 bond angle of 109.69 (9)° essentially equals the tetrahedral value of 109.5°. The C(methine)—P—C(aromatic) bond angles range from 102.67 (9) to 107.04 (9)°, whereas the C(aromatic)—P—C(aromatic) bond angles of 96.72 (9) and 97.29 (9)° are significantly smaller. The steric demands of the *o*-tolyl groups in C_58_H_58_P_4_ cause deviations from the bond lengths and angles reported for the related phenyl compound.

Like its phenyl analog, the title compound is chiral in the solid form, and its room temperature NMR spectra reveal that its chirality is retained in solution. The compound has an AA'BB' ^31^P spin system, and its methine protons are inequivalent in the ^1^H NMR spectrum.

### Experimental

The title compound, C_58_H_58_P_4_, is one of two isomeric products. Under an N_2_ atmosphere, *n*-BuLi (0.63 ml of a 1.6 *M* solution in hexanes, 1.0 mmol) was added over 1 h to a solution of bis(di-*o*-tolylphosphino)methane (0.44 g, 1.0 mmol) in toluene (5 ml). The solution was refluxed for 1 h and the solvent was removed under vacuum. The solid residue was redissolved in THF (5 ml) and added over 1 h to a suspension of PdCl_2_ in toluene (6 ml). The mixture was stirred overnight and the solvent was removed under vacuum. Toluene (8 ml) and pentane (50 ml) were added to dissolve the residue, and the mixture was filtered through Celite. The filtrate was concentrated to 3 ml and layered with pentane (15 ml). Yellow crystals of the title compound were obtained after 1 week at room temperature. ^1^H NMR (CDCl_3_): δ 1.65–2.18 (m, C_6_H_4_C*H*_3_), 3.27 (br s, C*H*), 4.55 (m, C*H*), 5.76 (br s, C_6_*H*_4_CH_3_), 6.05 (br s, C_6_*H*_4_CH_3_), 6.81–7.55 (m, C_6_*H*_4_CH_3_), 8.23 (br s, C_6_*H*_4_CH_3_). ^31^P {^1^H} NMR (CDCl_3_): δ -16.3 (m, P_A_), -39.3 (m, P_B_) of an AA'BB' spin system.

### Refinement

A structural model consisting of the molecule was developed using the Bruker *SHELXTL* suite of programs. Most of the non-hydrogen containing atoms were found in the E-map generated from the direct-methods solution. The remaining non-hydrogen atoms were located after full-matrix least squares / difference Fourier cycles were performed. All non-hydrogen atoms were refined with anisotropic displacement parameters. Methyl H atom positions, R—CH_3_, were optimized by rotation about R—C bonds with idealized C—H, R—H and H···H distances. Remaining H atoms were included as riding idealized contributors. Methyl H atom U's were assigned as 1.5 times *U*_eq_ of the carrier atom; remaining H atom U's were assigned as 1.2 times carrier *U*_eq_.

### Crystal data

**Table:**

C_58_H_58_P_4_	*F*(000) = 1864
*M**_r_* = 878.92	*D*_x_ = 1.235 Mg m^−^^3^
Monoclinic, *C*2/*c*	Mo *K*α radiation, λ = 0.71073 Å
Hall symbol: -C 2yc	Cell parameters from 3541 reflections
*a* = 21.8875 (11) Å	θ = 2.8–26.4°
*b* = 10.9702 (6) Å	µ = 0.20 mm^−^^1^
*c* = 19.691 (1) Å	*T* = 193 K
β = 90.761 (3)°	Prism, yellow
*V* = 4727.6 (4) Å^3^	0.42 × 0.40 × 0.24 mm
*Z* = 4	

### Data collection

**Table:**

Bruker Kappa APEXII CCD diffractometer	4345 independent reflections
Radiation source: fine-focus sealed tube	3343 reflections with *I* > 2σ(*I*)
graphite	*R*_int_ = 0.049
φ and ω scans	θ_max_ = 25.4°, θ_min_ = 1.9°
Absorption correction: multi-scan (*SADABS*; Bruker, 2005)	*h* = −26→26
*T*_min_ = 0.942, *T*_max_ = 0.976	*k* = −13→11
19717 measured reflections	*l* = −21→23

### Refinement

**Table:**

Refinement on *F*^2^	Primary atom site location: structure-invariant direct methods
Least-squares matrix: full	Secondary atom site location: difference Fourier map
*R*[*F*^2^ > 2σ(*F*^2^)] = 0.039	Hydrogen site location: inferred from neighbouring sites
*wR*(*F*^2^) = 0.102	H-atom parameters not refined
*S* = 1.01	*w* = 1/[σ^2^(*F*_o_^2^) + (0.0477*P*)^2^ + 3.4164*P*] where *P* = (*F*_o_^2^ + 2*F*_c_^2^)/3
4345 reflections	(Δ/σ)_max_ = 0.001
284 parameters	Δρ_max_ = 0.35 e Å^−^^3^
0 restraints	Δρ_min_ = −0.19 e Å^−^^3^

### Special details

**Table:**

Experimental. One distinct cell was identified using *APEX2* (Bruker, 2004). Four frame series were integrated and filtered for statistical outliers using *SAINT* (Bruker, 2005) then corrected for absorption by integration using *SHELXTL*/*XPREP* V2005/2 (Bruker, 2005) before using *SAINT*/*SADABS* (Bruker, 2005) to sort, merge,and scale the combined data. No decay correction was applied.
Geometry. All e.s.d.'s (except the e.s.d. in the dihedral angle between two l.s. planes) are estimated using the full covariance matrix. The cell e.s.d.'s are taken into account individually in the estimation of e.s.d.'s in distances, angles and torsion angles; correlations between e.s.d.'s in cell parameters are only used when they are defined by crystal symmetry. An approximate (isotropic) treatment of cell e.s.d.'s is used for estimating e.s.d.'s involving l.s. planes.
Refinement. Structure was phased by direct methods (Sheldrick, 2008). Systematic conditions suggested the ambiguous space group. The space group choice was confirmed by successful convergence of the full-matrix least-squares refinement on *F*^2^. The highest peaks in the final difference Fourier map were in the vicinity of atoms P1 and P2; the final map had no other significant features. A final analysis of variance between observed and calculated structure factors showed some dependence on amplitude but little on resolution.

### Fractional atomic coordinates and isotropic or equivalent isotropic displacement parameters (Å^2^)

**Table:**

	*x*	*y*	*z*	*U*_iso_*/*U*_eq_	
P1	0.39722 (2)	0.68981 (5)	0.23551 (3)	0.02565 (14)	
P2	0.48535 (2)	0.83242 (5)	0.33033 (2)	0.02367 (14)	
C1	0.47465 (8)	0.68809 (17)	0.27827 (9)	0.0222 (4)	
H1A	0.4790	0.6145	0.3079	0.027*	
C2	0.39830 (9)	0.54854 (18)	0.18376 (10)	0.0280 (5)	
C3	0.36720 (9)	0.5468 (2)	0.12096 (10)	0.0308 (5)	
C4	0.36966 (10)	0.4409 (2)	0.08185 (11)	0.0401 (6)	
H4A	0.3490	0.4391	0.0391	0.048*	
C5	0.40112 (11)	0.3389 (2)	0.10330 (12)	0.0433 (6)	
H5A	0.4026	0.2686	0.0752	0.052*	
C6	0.43039 (11)	0.3389 (2)	0.16554 (12)	0.0414 (6)	
H6A	0.4516	0.2685	0.1811	0.050*	
C7	0.42855 (10)	0.44320 (19)	0.20526 (11)	0.0345 (5)	
H7A	0.4485	0.4429	0.2484	0.041*	
C8	0.33175 (10)	0.6547 (2)	0.09454 (11)	0.0376 (5)	
H8A	0.3234	0.6441	0.0459	0.056*	
H8B	0.3557	0.7292	0.1018	0.056*	
H8C	0.2931	0.6612	0.1188	0.056*	
C9	0.34259 (9)	0.63472 (19)	0.30004 (10)	0.0305 (5)	
C10	0.28290 (10)	0.6830 (2)	0.30017 (11)	0.0385 (6)	
C11	0.24123 (11)	0.6334 (3)	0.34590 (13)	0.0513 (7)	
H11A	0.2008	0.6650	0.3466	0.062*	
C12	0.25671 (12)	0.5408 (3)	0.38986 (13)	0.0561 (7)	
H12A	0.2272	0.5089	0.4200	0.067*	
C13	0.31522 (12)	0.4946 (2)	0.38997 (12)	0.0480 (6)	
H13A	0.3265	0.4312	0.4205	0.058*	
C14	0.35750 (10)	0.5414 (2)	0.34532 (11)	0.0369 (5)	
H14A	0.3978	0.5090	0.3455	0.044*	
C15	0.26167 (11)	0.7842 (2)	0.25435 (13)	0.0495 (6)	
H15A	0.2318	0.8346	0.2781	0.074*	
H15B	0.2427	0.7496	0.2133	0.074*	
H15C	0.2967	0.8345	0.2417	0.074*	
C16	0.40878 (9)	0.87239 (19)	0.36281 (10)	0.0276 (4)	
C17	0.37778 (10)	0.9723 (2)	0.33442 (11)	0.0353 (5)	
C18	0.32210 (11)	1.0061 (2)	0.36232 (12)	0.0470 (6)	
H18A	0.3005	1.0731	0.3430	0.056*	
C19	0.29730 (11)	0.9465 (2)	0.41646 (13)	0.0481 (7)	
H19A	0.2592	0.9722	0.4341	0.058*	
C20	0.32791 (10)	0.8488 (2)	0.44523 (12)	0.0389 (5)	
H20A	0.3112	0.8068	0.4828	0.047*	
C21	0.38290 (9)	0.8131 (2)	0.41877 (10)	0.0311 (5)	
H21A	0.4040	0.7463	0.4389	0.037*	
C22	0.40310 (13)	1.0441 (2)	0.27621 (13)	0.0538 (7)	
H22A	0.3741	1.1083	0.2631	0.081*	
H22B	0.4097	0.9897	0.2375	0.081*	
H22C	0.4420	1.0812	0.2901	0.081*	
C23	0.52007 (8)	0.78697 (18)	0.41210 (9)	0.0244 (4)	
C24	0.55118 (9)	0.87983 (19)	0.44804 (10)	0.0307 (5)	
C25	0.57354 (11)	0.8554 (2)	0.51278 (11)	0.0395 (6)	
H25A	0.5939	0.9182	0.5374	0.047*	
C26	0.56695 (11)	0.7426 (2)	0.54230 (11)	0.0410 (6)	
H26A	0.5822	0.7284	0.5870	0.049*	
C27	0.53823 (10)	0.6504 (2)	0.50693 (11)	0.0385 (5)	
H27A	0.5343	0.5718	0.5267	0.046*	
C28	0.51505 (9)	0.67275 (19)	0.44232 (10)	0.0308 (5)	
H28A	0.4953	0.6087	0.4181	0.037*	
C29	0.56121 (12)	1.0050 (2)	0.41859 (12)	0.0467 (6)	
H29A	0.5803	1.0576	0.4530	0.070*	
H29B	0.5218	1.0399	0.4044	0.070*	
H29C	0.5880	0.9989	0.3792	0.070*	

### Atomic displacement parameters (Å^2^)

**Table:**

	*U*^11^	*U*^22^	*U*^33^	*U*^12^	*U*^13^	*U*^23^
P1	0.0244 (3)	0.0252 (3)	0.0273 (3)	−0.0011 (2)	−0.0027 (2)	−0.0013 (2)
P2	0.0255 (3)	0.0219 (3)	0.0236 (3)	0.0003 (2)	−0.0008 (2)	−0.0012 (2)
C1	0.0225 (10)	0.0212 (10)	0.0230 (9)	0.0001 (8)	0.0004 (8)	0.0001 (8)
C2	0.0265 (10)	0.0289 (11)	0.0286 (11)	−0.0079 (9)	0.0040 (8)	−0.0023 (9)
C3	0.0264 (11)	0.0381 (12)	0.0279 (11)	−0.0111 (9)	0.0049 (8)	−0.0030 (9)
C4	0.0375 (13)	0.0503 (15)	0.0325 (12)	−0.0131 (11)	0.0014 (10)	−0.0090 (11)
C5	0.0476 (14)	0.0360 (14)	0.0467 (14)	−0.0114 (11)	0.0092 (11)	−0.0157 (11)
C6	0.0487 (14)	0.0270 (12)	0.0486 (14)	−0.0040 (11)	0.0087 (11)	−0.0049 (11)
C7	0.0402 (13)	0.0300 (12)	0.0335 (12)	−0.0061 (10)	0.0035 (9)	−0.0025 (9)
C8	0.0323 (12)	0.0480 (14)	0.0325 (11)	−0.0062 (11)	−0.0042 (9)	−0.0013 (10)
C9	0.0270 (11)	0.0340 (12)	0.0305 (11)	−0.0058 (9)	0.0004 (9)	−0.0101 (9)
C10	0.0279 (11)	0.0482 (15)	0.0393 (12)	−0.0022 (10)	−0.0013 (9)	−0.0190 (11)
C11	0.0289 (12)	0.077 (2)	0.0488 (15)	−0.0046 (13)	0.0070 (11)	−0.0227 (15)
C12	0.0441 (15)	0.078 (2)	0.0463 (15)	−0.0204 (15)	0.0180 (12)	−0.0119 (15)
C13	0.0509 (15)	0.0532 (16)	0.0401 (13)	−0.0119 (13)	0.0099 (11)	−0.0006 (12)
C14	0.0344 (12)	0.0395 (13)	0.0368 (12)	−0.0050 (10)	0.0058 (10)	−0.0041 (10)
C15	0.0350 (13)	0.0583 (17)	0.0550 (15)	0.0100 (12)	−0.0064 (11)	−0.0154 (13)
C16	0.0265 (10)	0.0278 (11)	0.0283 (10)	0.0020 (9)	−0.0047 (8)	−0.0078 (9)
C17	0.0386 (12)	0.0362 (13)	0.0310 (11)	0.0101 (10)	−0.0051 (9)	−0.0064 (10)
C18	0.0418 (14)	0.0529 (16)	0.0460 (14)	0.0241 (12)	−0.0066 (11)	−0.0060 (12)
C19	0.0307 (13)	0.0646 (18)	0.0491 (15)	0.0133 (12)	0.0015 (11)	−0.0127 (13)
C20	0.0297 (12)	0.0483 (15)	0.0387 (12)	−0.0013 (11)	0.0036 (10)	−0.0087 (11)
C21	0.0284 (11)	0.0323 (12)	0.0325 (11)	0.0027 (9)	−0.0001 (9)	−0.0067 (9)
C22	0.0684 (18)	0.0474 (16)	0.0456 (15)	0.0237 (14)	0.0029 (13)	0.0111 (12)
C23	0.0215 (10)	0.0274 (11)	0.0244 (10)	0.0041 (8)	0.0018 (8)	−0.0011 (8)
C24	0.0315 (11)	0.0307 (12)	0.0299 (11)	0.0024 (9)	−0.0028 (9)	−0.0066 (9)
C25	0.0444 (14)	0.0397 (14)	0.0343 (12)	0.0005 (11)	−0.0072 (10)	−0.0090 (11)
C26	0.0458 (14)	0.0513 (15)	0.0258 (11)	0.0071 (12)	−0.0086 (10)	−0.0026 (11)
C27	0.0453 (13)	0.0378 (13)	0.0324 (12)	0.0023 (11)	−0.0007 (10)	0.0067 (10)
C28	0.0327 (11)	0.0311 (12)	0.0284 (10)	−0.0019 (9)	−0.0011 (9)	−0.0011 (9)
C29	0.0640 (16)	0.0334 (13)	0.0422 (14)	−0.0106 (12)	−0.0127 (12)	−0.0073 (11)

### Geometric parameters (Å, °)

**Table:**

P1—C2	1.855 (2)	C14—H14A	0.9500
P1—C9	1.857 (2)	C15—H15A	0.9800
P1—C1	1.8824 (19)	C15—H15B	0.9800
P2—C23	1.8401 (19)	C15—H15C	0.9800
P2—C16	1.854 (2)	C16—C17	1.402 (3)
P2—C1	1.8991 (19)	C16—C21	1.405 (3)
C1—C1^i^	1.582 (4)	C17—C18	1.394 (3)
C1—H1A	1.0000	C17—C22	1.502 (3)
C2—C7	1.395 (3)	C18—C19	1.369 (3)
C2—C3	1.404 (3)	C18—H18A	0.9500
C3—C4	1.396 (3)	C19—C20	1.381 (3)
C3—C8	1.504 (3)	C19—H19A	0.9500
C4—C5	1.377 (3)	C20—C21	1.375 (3)
C4—H4A	0.9500	C20—H20A	0.9500
C5—C6	1.375 (3)	C21—H21A	0.9500
C5—H5A	0.9500	C22—H22A	0.9800
C6—C7	1.387 (3)	C22—H22B	0.9800
C6—H6A	0.9500	C22—H22C	0.9800
C7—H7A	0.9500	C23—C28	1.392 (3)
C8—H8A	0.9800	C23—C24	1.410 (3)
C8—H8B	0.9800	C24—C25	1.385 (3)
C8—H8C	0.9800	C24—C29	1.508 (3)
C9—C14	1.394 (3)	C25—C26	1.376 (3)
C9—C10	1.410 (3)	C25—H25A	0.9500
C10—C11	1.400 (3)	C26—C27	1.375 (3)
C10—C15	1.501 (3)	C26—H26A	0.9500
C11—C12	1.374 (4)	C27—C28	1.385 (3)
C11—H11A	0.9500	C27—H27A	0.9500
C12—C13	1.377 (4)	C28—H28A	0.9500
C12—H12A	0.9500	C29—H29A	0.9800
C13—C14	1.383 (3)	C29—H29B	0.9800
C13—H13A	0.9500	C29—H29C	0.9800
			
C2—P1—C9	96.72 (9)	C10—C15—H15A	109.5
C2—P1—C1	102.67 (9)	C10—C15—H15B	109.5
C9—P1—C1	105.95 (9)	H15A—C15—H15B	109.5
C23—P2—C16	97.29 (9)	C10—C15—H15C	109.5
C23—P2—C1	107.04 (9)	H15A—C15—H15C	109.5
C16—P2—C1	106.12 (8)	H15B—C15—H15C	109.5
C1^i^—C1—P1	108.72 (16)	C17—C16—C21	118.48 (19)
C1^i^—C1—P2	107.29 (9)	C17—C16—P2	118.84 (16)
P1—C1—P2	109.69 (9)	C21—C16—P2	122.44 (15)
C1^i^—C1—H1A	110.4	C18—C17—C16	118.2 (2)
P1—C1—H1A	110.4	C18—C17—C22	119.7 (2)
P2—C1—H1A	110.4	C16—C17—C22	122.2 (2)
C7—C2—C3	118.62 (19)	C19—C18—C17	122.5 (2)
C7—C2—P1	122.33 (15)	C19—C18—H18A	118.7
C3—C2—P1	119.05 (16)	C17—C18—H18A	118.7
C4—C3—C2	118.4 (2)	C18—C19—C20	119.7 (2)
C4—C3—C8	119.20 (19)	C18—C19—H19A	120.2
C2—C3—C8	122.41 (19)	C20—C19—H19A	120.2
C5—C4—C3	122.0 (2)	C21—C20—C19	119.2 (2)
C5—C4—H4A	119.0	C21—C20—H20A	120.4
C3—C4—H4A	119.0	C19—C20—H20A	120.4
C6—C5—C4	119.9 (2)	C20—C21—C16	121.9 (2)
C6—C5—H5A	120.1	C20—C21—H21A	119.0
C4—C5—H5A	120.1	C16—C21—H21A	119.0
C5—C6—C7	119.1 (2)	C17—C22—H22A	109.5
C5—C6—H6A	120.4	C17—C22—H22B	109.5
C7—C6—H6A	120.4	H22A—C22—H22B	109.5
C6—C7—C2	122.0 (2)	C17—C22—H22C	109.5
C6—C7—H7A	119.0	H22A—C22—H22C	109.5
C2—C7—H7A	119.0	H22B—C22—H22C	109.5
C3—C8—H8A	109.5	C28—C23—C24	118.42 (18)
C3—C8—H8B	109.5	C28—C23—P2	125.72 (15)
H8A—C8—H8B	109.5	C24—C23—P2	115.75 (15)
C3—C8—H8C	109.5	C25—C24—C23	119.0 (2)
H8A—C8—H8C	109.5	C25—C24—C29	118.57 (19)
H8B—C8—H8C	109.5	C23—C24—C29	122.45 (18)
C14—C9—C10	118.9 (2)	C26—C25—C24	121.6 (2)
C14—C9—P1	121.96 (16)	C26—C25—H25A	119.2
C10—C9—P1	118.96 (17)	C24—C25—H25A	119.2
C11—C10—C9	117.9 (2)	C27—C26—C25	119.8 (2)
C11—C10—C15	118.3 (2)	C27—C26—H26A	120.1
C9—C10—C15	123.8 (2)	C25—C26—H26A	120.1
C12—C11—C10	122.3 (2)	C26—C27—C28	119.6 (2)
C12—C11—H11A	118.8	C26—C27—H27A	120.2
C10—C11—H11A	118.8	C28—C27—H27A	120.2
C11—C12—C13	119.6 (2)	C27—C28—C23	121.5 (2)
C11—C12—H12A	120.2	C27—C28—H28A	119.3
C13—C12—H12A	120.2	C23—C28—H28A	119.3
C12—C13—C14	119.5 (3)	C24—C29—H29A	109.5
C12—C13—H13A	120.2	C24—C29—H29B	109.5
C14—C13—H13A	120.2	H29A—C29—H29B	109.5
C13—C14—C9	121.7 (2)	C24—C29—H29C	109.5
C13—C14—H14A	119.1	H29A—C29—H29C	109.5
C9—C14—H14A	119.1	H29B—C29—H29C	109.5
			
C2—P1—C1—C1^i^	59.35 (7)	C11—C12—C13—C14	−0.6 (4)
C9—P1—C1—C1^i^	160.24 (7)	C12—C13—C14—C9	0.2 (4)
C2—P1—C1—P2	176.38 (9)	C10—C9—C14—C13	0.3 (3)
C9—P1—C1—P2	−82.73 (11)	P1—C9—C14—C13	−175.23 (18)
C23—P2—C1—C1^i^	−105.45 (15)	C23—P2—C16—C17	142.76 (16)
C16—P2—C1—C1^i^	151.44 (14)	C1—P2—C16—C17	−107.09 (16)
C23—P2—C1—P1	136.62 (9)	C23—P2—C16—C21	−31.53 (18)
C16—P2—C1—P1	33.51 (12)	C1—P2—C16—C21	78.62 (18)
C9—P1—C2—C7	−73.92 (18)	C21—C16—C17—C18	−1.4 (3)
C1—P1—C2—C7	34.14 (19)	P2—C16—C17—C18	−175.89 (17)
C9—P1—C2—C3	105.52 (16)	C21—C16—C17—C22	177.7 (2)
C1—P1—C2—C3	−146.42 (15)	P2—C16—C17—C22	3.2 (3)
C7—C2—C3—C4	−2.1 (3)	C16—C17—C18—C19	0.8 (4)
P1—C2—C3—C4	178.47 (15)	C22—C17—C18—C19	−178.3 (2)
C7—C2—C3—C8	178.14 (19)	C17—C18—C19—C20	−0.1 (4)
P1—C2—C3—C8	−1.3 (3)	C18—C19—C20—C21	−0.1 (4)
C2—C3—C4—C5	0.5 (3)	C19—C20—C21—C16	−0.6 (3)
C8—C3—C4—C5	−179.7 (2)	C17—C16—C21—C20	1.3 (3)
C3—C4—C5—C6	1.2 (3)	P2—C16—C21—C20	175.60 (17)
C4—C5—C6—C7	−1.1 (3)	C16—P2—C23—C28	83.90 (18)
C5—C6—C7—C2	−0.5 (3)	C1—P2—C23—C28	−25.49 (19)
C3—C2—C7—C6	2.2 (3)	C16—P2—C23—C24	−92.16 (16)
P1—C2—C7—C6	−178.40 (16)	C1—P2—C23—C24	158.45 (15)
C2—P1—C9—C14	67.02 (18)	C28—C23—C24—C25	−2.3 (3)
C1—P1—C9—C14	−38.25 (19)	P2—C23—C24—C25	174.03 (16)
C2—P1—C9—C10	−108.55 (17)	C28—C23—C24—C29	177.5 (2)
C1—P1—C9—C10	146.18 (16)	P2—C23—C24—C29	−6.1 (3)
C14—C9—C10—C11	−0.4 (3)	C23—C24—C25—C26	1.0 (3)
P1—C9—C10—C11	175.27 (16)	C29—C24—C25—C26	−178.9 (2)
C14—C9—C10—C15	179.4 (2)	C24—C25—C26—C27	0.8 (4)
P1—C9—C10—C15	−4.9 (3)	C25—C26—C27—C28	−1.3 (3)
C9—C10—C11—C12	0.0 (3)	C26—C27—C28—C23	−0.1 (3)
C15—C10—C11—C12	−179.8 (2)	C24—C23—C28—C27	1.9 (3)
C10—C11—C12—C13	0.5 (4)	P2—C23—C28—C27	−174.07 (16)

Symmetry codes: (i) −*x*+1, *y*, −*z*+1/2.
